# Supplementary figures and images for: Heterozygous deletion of Gpr55 does not affect a hyperthermia-induced seizure, spontaneous seizures or survival in the Scn1a+/- mouse model of Dravet syndrome
Source: PLoS One. 2023 Jan 26;18(1):e0280842. doi: 10.1371/journal.pone.0280842 (PMC9879440; doi:10.1371/journal.pone.0280842)

**A**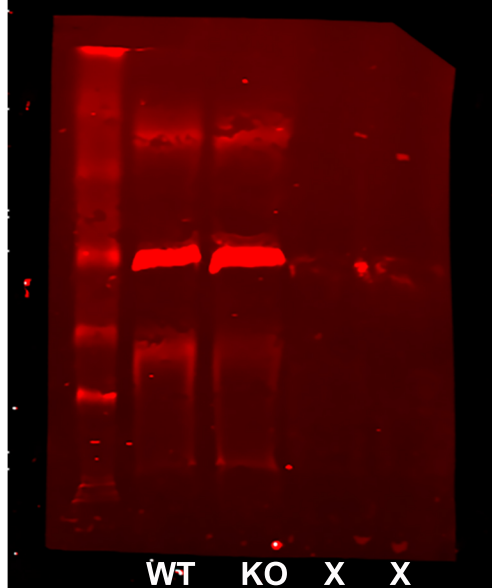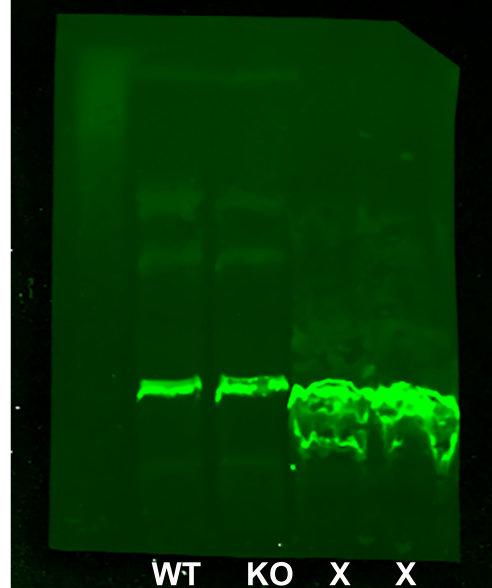**B**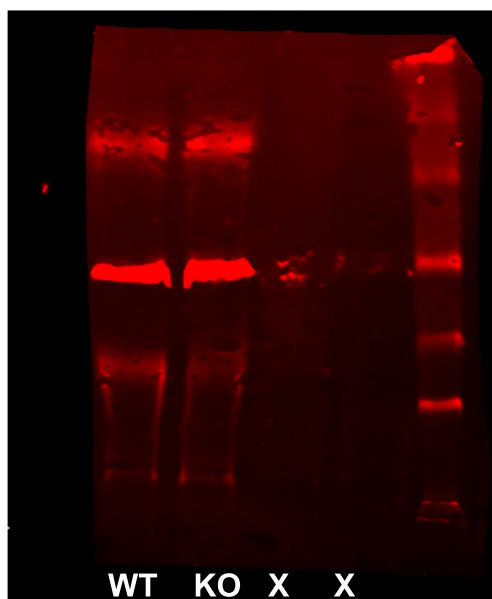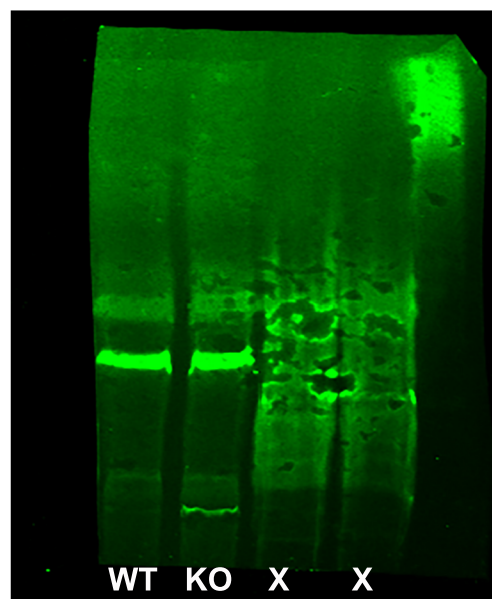**C**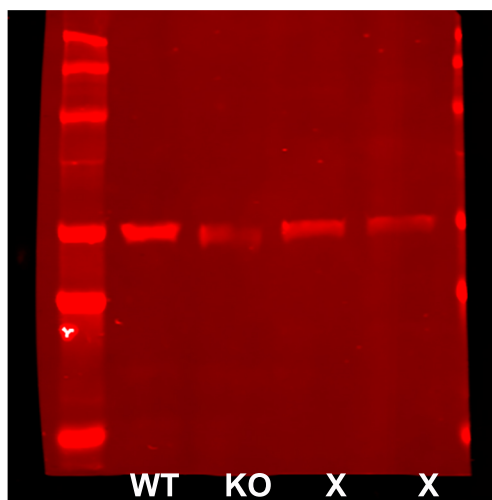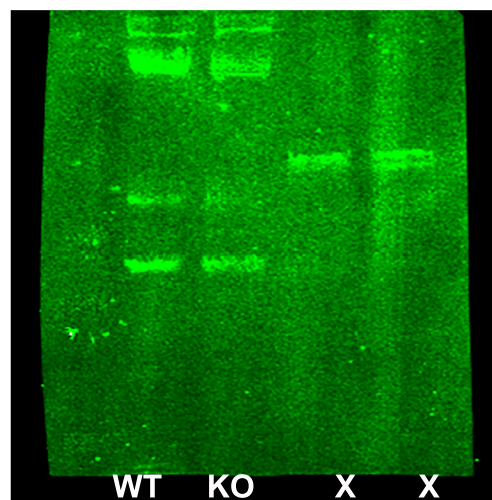**D**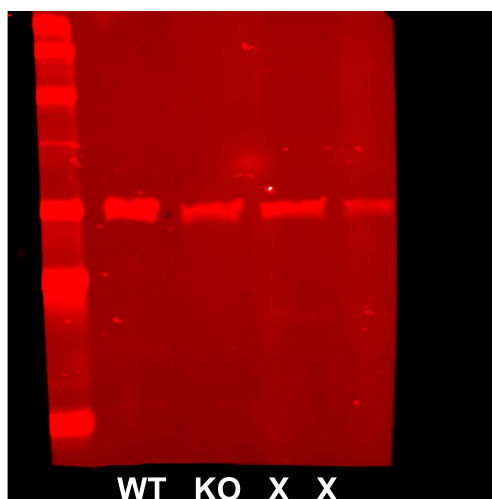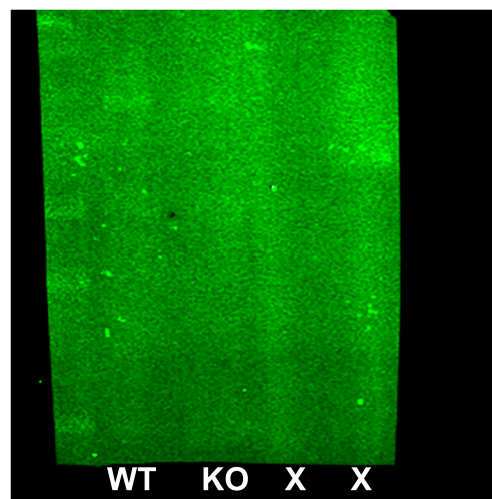

**S1 Fig.**  
**Raw images**

Supplement: S1 Fig — Western blot analysis of Gpr55 receptor levels in whole brain membrane preparations from wildtype (WT) and Gpr55-/- (KO) mice using (A) ThermoFisher, (B) Abcam and (C) Cayman Chemical primary GPR55 antibodies (right panels) with β-actin or β-tubulin serving as loading controls (left panels). None of these antibodies appear to be selective for mouse Gpr55. Precision Plus Protein Kaleidoscope ladder (Bio-Rad Laboratories). (D) Western blot analysis using the Cayman chemical GPR55 antibody blocked with a GPR55 blocking peptide. (PDF) [file pone.0280842.s001.pdf]
