# Supplementary material for: Heterozygous deletion of Gpr55 does not affect a hyperthermia-induced seizure, spontaneous seizures or survival in the Scn1a+/- mouse model of Dravet syndrome
Source: PLoS One. 2023 Jan 26;18(1):e0280842. doi: 10.1371/journal.pone.0280842 (PMC9879440; doi:10.1371/journal.pone.0280842)
Supplement: S1 File — (DOCX) [file pone.0280842.s003.DOCX]

**Supporting information**

**S1 Fig. Commercially-available GPR55 antibodies.** Western blot analysis of Gpr55 receptor levels in whole brain membrane preparations from wildtype (WT) and *Gpr55*^-/-^ (KO) mice using (**A**) ThermoFisher, (**B**) Abcam and (**C**) Cayman Chemical primary GPR55 antibodies (right panels) with β-actin or β-tubulin serving as loading controls (left panels). None of these enzymes appear to be selective for mouse Gpr55. Precision Plus Protein Kaleidoscope ladder (Bio-Rad Laboratories). (**D**) Western blot analysis using the Cayman chemical GPR55 antibody blocked with a GPR55 blocking peptide.
